# Supplementary material for: Practical considerations for measuring the effective reproductive number, Rt
Source: medRxiv. 2020 Aug 28:2020.06.18.20134858. Originally published 2020 Jun 20. Preprint. [Version 3] doi: 10.1101/2020.06.18.20134858 (PMC7325187; doi:10.1101/2020.06.18.20134858)
Supplement: 1 [file NIHPP2020.06.18.20134858-supplement-1.pdf]

medRxiv preprint doi: https://doi.org/10.1101/2020.06.18.20134858.this version posted August 28, 2020. The copyright holder for this preprint
(which was not certified by peer review) is the author/funder, who has granted medRxiv a license to display the preprint in perpetuity.
It is made available under a CC-BY 4.0 International license .

Supporting information

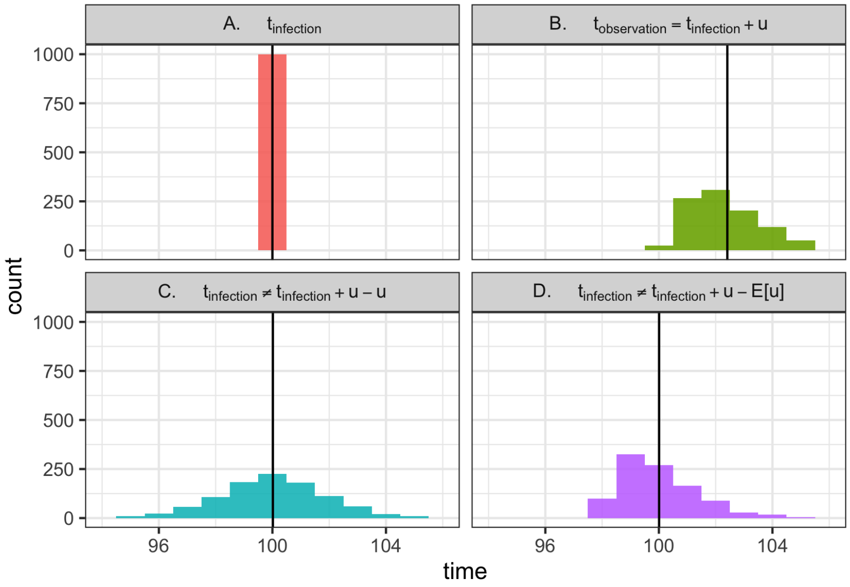

S1 Fig. Why is deconvolution needed to recover latent times of infection? (A) Consider 1000 individuals, all infected at time 100. (Vertical line shows the mean). (B) Now consider the times at which these individuals are observed. Logically, $t_{\text{observation}} = t_{\text{infected}} + u$, where u is a random variable describing the delay between infection and observation. Mathematically, this is a convolution of the infection time and the delay distribution. Because u has non-zero variance, observation times are not only shifted into the future but also are blurred across many dates. This blurring is biologically realistic; due to variability in disease progression and care seeking, individuals with the same date of infection will not necessarily be observed at the same time. (C) Using the observations in B, we aim to recover the latent times of infection shown in A. Doing so would require not only shifting into the past but also removing the variance introduced by the observation process, which can be achieved by deconvolution. Instead, as demonstrated here, a common strategy is to subtract u from the times of observation, effectively repeating the convolution shown in B, but this time moving backward in time rather than forward. This is not the correct inverse operation. It fails to remove variance introduced by the observation process (the forward convolution) and adds new, biologically unrealistic variance, further blurring the inferred times of infection. (D) Shifting the times of observation by the mean delay $E[u]$ is also incorrect, as it does not remove the variance from the forward convolution in B. But if the mean delay time is known exactly, this approach is preferable to C, as it avoids adding even more variance. Ultimately, deconvolution methods would be needed to recover A from the observations in B while properly accounting for uncertainty.

medRxiv preprint doi: https://doi.org/10.1101/2020.06.18.20134858.this version posted August 28, 2020. The copyright holder for this preprint
(which was not certified by peer review) is the author/funder, who has granted medRxiv a license to display the preprint in perpetuity.
It is made available under a CC-BY 4.0 International license .

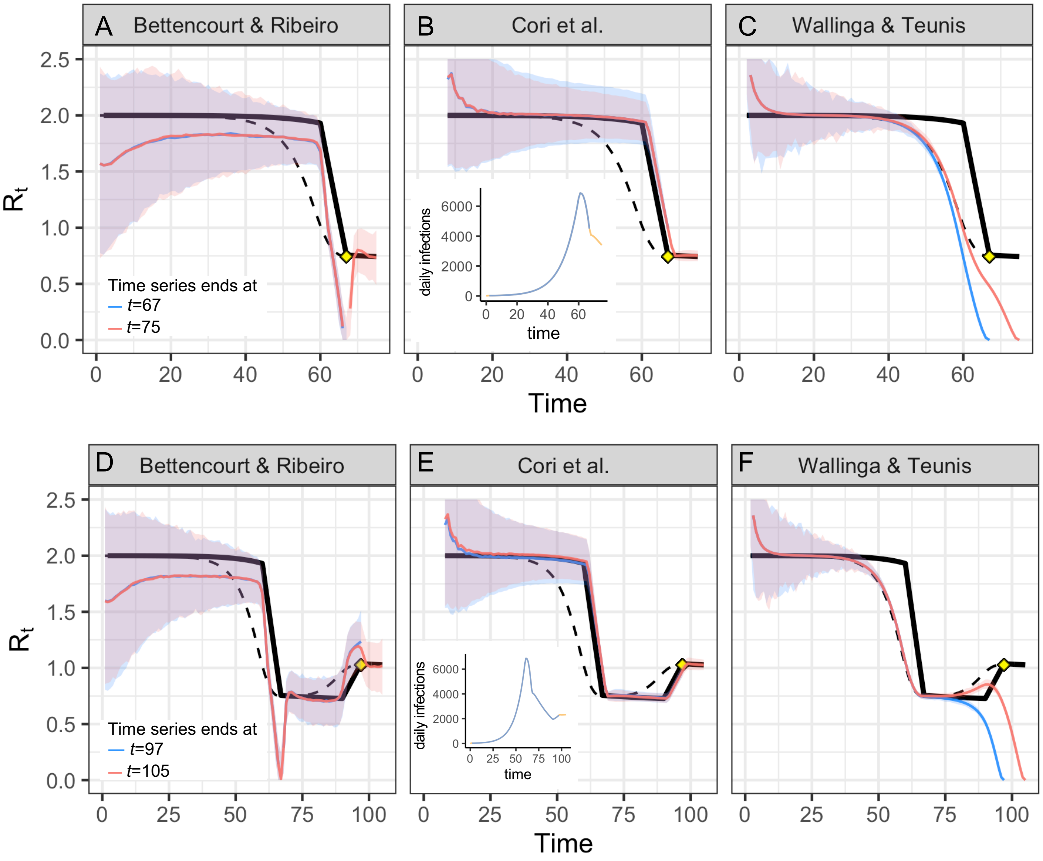

S2 Fig. real-time accuracy when R_t is rising or falling. (A-C) Alternate version of Fig. 2 in which the time series ends on the day R_t first hits its minimum value after falling abruptly (time 67, yellow point), or eight days after the changepoint (time 75). (D-F) The time series ends on the day R_t stops rising (time 97, yellow point), or eight days later (time 105). Estimates of the instantaneous reproductive number (A,B,D,E) remain accurate to the end of the time series, and estimates do not change as new observations become available in the 8 days following the changepoint. As in the main text, estimates of the unadjusted case reproductive number (C,F) depend on data from not-yet-observed time points. These estimates become more accurate as new observations are added to the end of the time series (orange vs. blue). Methods to infer the number of not-yet-observed infections can help make estimates of the case reproductive number more accurate in real-time [4, 30]. All panels show fits to the time series of new infections, and assume all infections are observed instantaneously. Solid black line shows the instantaneous reproductive number, and dashed black line shows the case reproductive number. Colored lines and confidence region show posterior mean and 95% credible interval (A,B,D,E) or maximum likelihood estimate and 95% confidence interval (C,F).

medRxiv preprint doi: https://doi.org/10.1101/2020.06.18.20134858.this version posted August 28, 2020. The copyright holder for this preprint (which was not certified by peer review) is the author/funder, who has granted medRxiv a license to display the preprint in perpetuity.
It is made available under a CC-BY 4.0 International license .

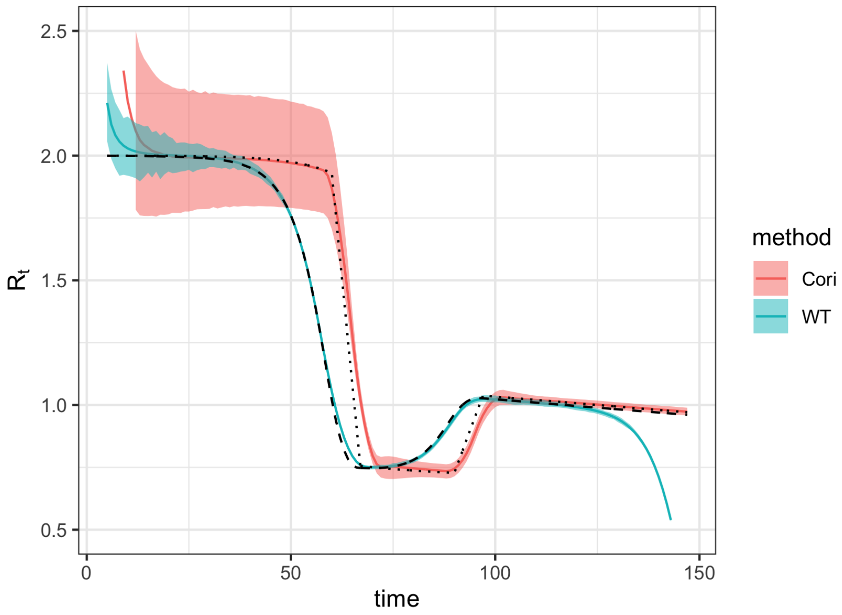

S3 Fig. Smoothed estimates of Cori et al. and Wallinga and Teunis. Both were estimated using a 7-day smoothing window on a synthetic time series of new infections, observed without delay. The estimates of Cori et al. and Wallinga and Teunis are similar in shape when smoothed, but the estimate of Wallinga and Teunis (the case reproductive number) leads that of Cori et al. (the instantaneous reproductive number) by roughly 8 days, or the mean generation interval. Solid colored lines and confidence regions show the posterior mean and 95% credible interval (Cori et al.) or maximum likelihood estimate and 95% confidence interval (Wallinga and Teunis). Dotted and dashed lines show the exact instantaneous reproductive number and case reproductive number, respectively.
